# Supplementary material for: Strain-level metagenomic assignment and compositional estimation for long reads with MetaMaps
Source: Nat Commun. 2019 Jul 11;10:3066. doi: 10.1038/s41467-019-10934-2 (PMC6624308; doi:10.1038/s41467-019-10934-2)
Supplement: Supplementary file 1 — Supplementary Information [file 41467_2019_10934_MOESM1_ESM.pdf]

Supplementary Information

Strain-level metagenomic assignment and compositional estimation for long reads with MetaMaps

Dilthey et al.

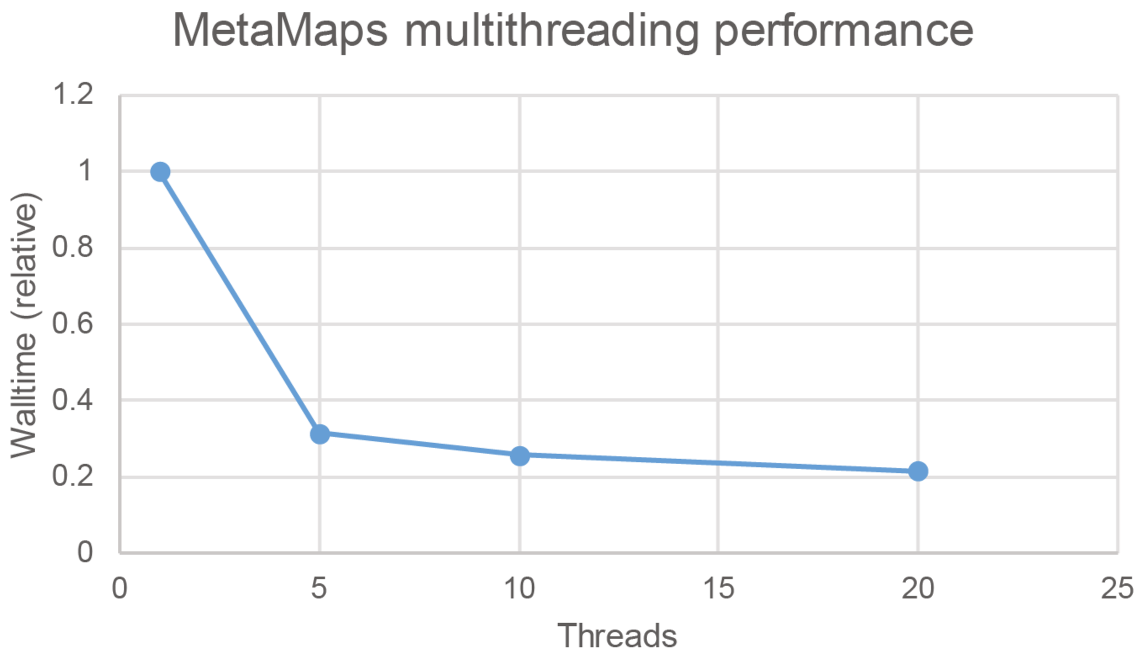

Supplementary Figure 1. MetaMaps multithreading performance. Relative runtime (wall time) of MetaMaps, measured on HMP7 data, depending on the number of utilized CPU cores.

Reads	Reads %		COG group
390	0.14%	A	RNA processing and modification
399	0.15%	B	Chromatin structure and dynamics
47263	17.31%	C	Energy production and conversion
12889	4.72%	D	Cell cycle control, cell division, chromosome partitioning
68289	25.02%	E	Amino acid transport and metabolism
29636	10.86%	F	Nucleotide transport and metabolism
58230	21.33%	G	Carbohydrate transport and metabolism
31347	11.48%	H	Coenzyme transport and metabolism
26591	9.74%	I	Lipid transport and metabolism
49020	17.96%	J	Translation, ribosomal structure and biogenesis
70430	25.80%	K	Transcription
61699	22.60%	L	Replication, recombination and repair
59690	21.87%	M	Cell wall/membrane/envelope biogenesis
12514	4.58%	N	Cell motility
37550	13.76%	O	Post-translational modification, protein turnover, and chaperones
55657	20.39%	P	Inorganic ion transport and metabolism
13808	5.06%	Q	Secondary metabolites biosynthesis, transport, and catabolism
154932	56.76%	S	Function unknown
33782	12.38%	T	Signal transduction mechanisms
18251	6.69%	U	Intracellular trafficking, secretion, and vesicular transport
24103	8.83%	V	Defense mechanisms
408	0.15%	W	Extracellular structures
116	0.04%	Y	Nuclear structure
131	0.05%	Z	Cytoskeleton

Supplementary Figure 2. COG (Clusters of Orthologous Genes) analysis of the HMP7 data. HMP7 reads are mapped against a COG-annotated version of the MetaMaps database, and the number of reads overlapping with genes annotated with specific COG groups is tabulated.

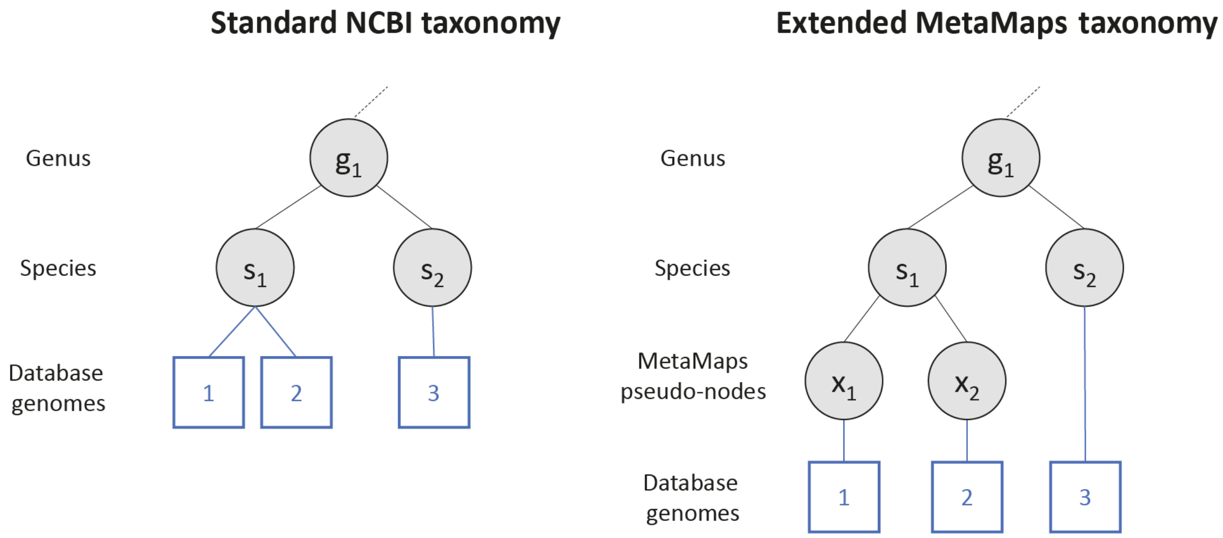

Supplementary Figure 3. The extended MetaMaps taxonomy. MetaMaps uses an extended version of the NCBI taxonomy in which each reference database genome has a unique taxon ID. This is constructed by creating additional pseudo taxon IDs (prefixed with an 'x'), which distinguish between genomes attached to the same node in the original NCBI taxonomy.

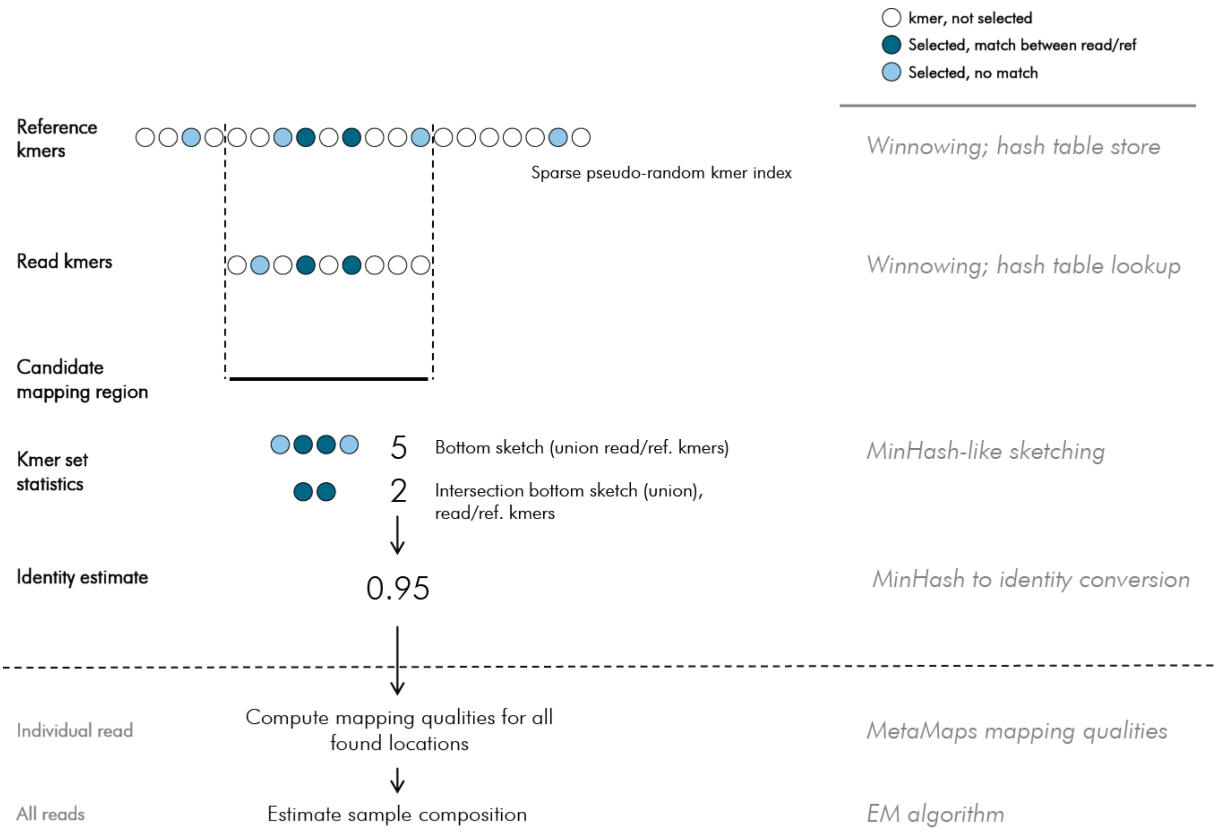

Supplementary Figure 4. High-level overview of the approximate mapping algorithm (MashMap). Minimizers are selected from the reference and from the reads. Minimizer matches between read and reference are identified using a hash table, inducing candidate mapping locations. Minimizer density is determined based on minimum read length and alignment identity. For each candidate mapping location, we use a winnowed-minhash approach, based on read and reference minimizers, to estimate the Jaccard similarity between the full kmer sets of the read and the candidate mapping location, and convert this estimate into an estimate of alignment identity. The steps below the dashed line show the subsequent steps of mapping quality computation and EM-based sample composition estimation.

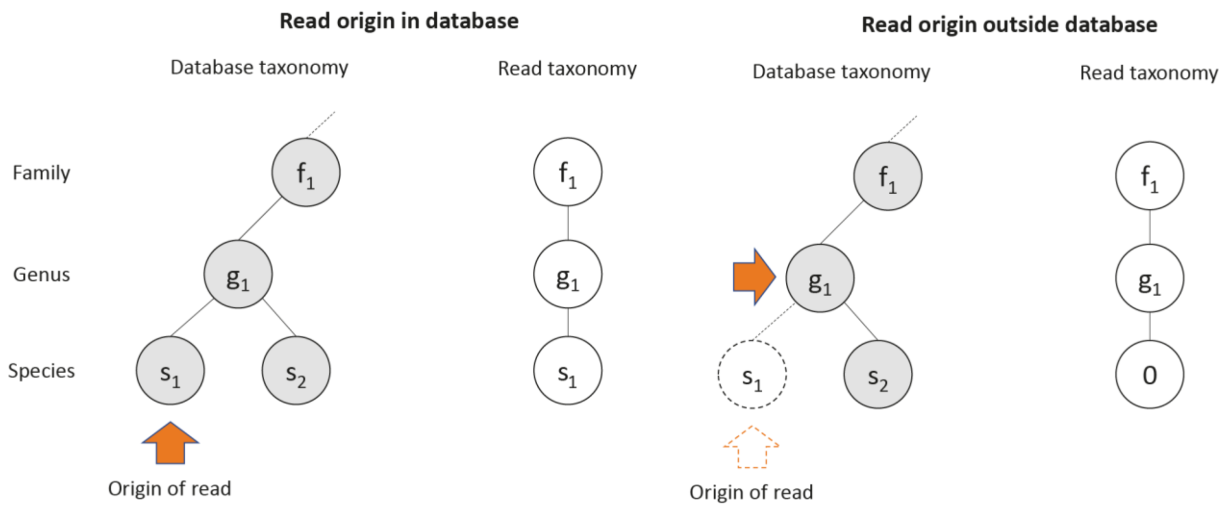

Supplementary Figure 5. Evaluation in the presence of out-of-database genomes. In some experiments, not all genomes present in the input data are present in the reference database. We assign reads that emanate from out-of-database entities to the taxonomic node that represents the most recent common ancestor of the read's source genome and its next-closest database relative; and for all taxonomic levels below the most-recent-common-ancestor node, true read assignment is defined as "Unassigned" (special taxon ID 0).

Experiment	Kraken/Bracken		Kraken2		Centrifuge		LAST+MEGAN-LR		MetaMaps	
	CPU hours	Peak memory (GB)	CPU hours	Peak memory (GB)	CPU hours	Peak memory (GB)	CPU hours	Peak memory (GB)	CPU hours	Peak memory (GB)
i100	0,20	154	0,05	21	0,32	11	6,01	20	16,64	262
p25	0,16	154	0,05	21	0,17	11	5,36	20	28,48	262
HMP7	0,25	154	0,15	21	0,21	11	7,65	20	30,80	262
Zymo	1,67	154	0,33	21	0,97	12	27,12	79	209,89	262
Cami	0,82	154	0,36	21	0,72	11	18,14	26	17,83	262
i100 (limited memory 20GB, MetaMaps only)	NA	NA	NA	NA	NA	NA	NA	NA	22,00	28
i100 (limited memory 10GB, MetaMaps only)	NA	NA	NA	NA	NA	NA	NA	NA	24,00	16
i100 (read length 2000, MetaMaps only)	NA	NA	NA	NA	NA	NA	NA	NA	15,23	139
HMP7 (limited memory 20GB, MetaMaps only)	NA	NA	NA	NA	NA	NA	NA	NA	36,75	28
HMP7 (limited memory 10GB, MetaMaps only, threads = 5)	NA	NA	NA	NA	NA	NA	NA	NA	40,89	16
HMP7 (read length 2000, MetaMaps only)	NA	NA	NA	NA	NA	NA	NA	NA	14,13	139

Supplementary Table 1. CPU time and peak memory on simulated and real data. The i100 “limited memory” experiment was run with a target maximum memory amount of 20GB (`--maxmemory 20`).
