# Supplementary material for: Strain-level metagenomic assignment and compositional estimation for long reads with MetaMaps
Source: Nat Commun. 2019 Jul 11;10:3066. doi: 10.1038/s41467-019-10934-2 (PMC6624308; doi:10.1038/s41467-019-10934-2)
Supplement: Supplementary file 4 — Description of Additional Supplementary Files [file 41467_2019_10934_MOESM4_ESM.pdf]

Description of Additional Supplementary Files

Supplementary Data 1. Read assignment accuracy. The table shows read assignment accuracy at different evaluation levels for the p25, i100, HMP7, Zymo, CAMI, and e2 ("Toxoplasma" and "Mosquito") experiments. Strain-level accuracy is measured at the level of individual database genomes (see Materials and methods). Kraken, Kraken 2 and LAST+MEGAN-LR don't support strain-level analysis and are therefore not validated at this level. "# Reads" specifies the total size of the input read set; "Precision" is the proportion of read assignments that are correct; "Precision2" is the proportion of non-0 read assignments that are correct; "Recall" is the proportion of reads having received a correct assignment.

Supplementary Data 2. Base-level assignment accuracy. The table shows base-level assignment accuracy at different evaluation levels for the p25, i100, HMP7, Zymo, CAMI, and e2 ("Toxoplasma" and "Mosquito") experiments. The metrics are equivalent to these reported in Supplementary Data 1, weighting each evaluated read by its length in bases.

Supplementary Data 3. Accuracy of compositional estimation. The table shows the accuracy of compositional estimation at different evaluation levels for the p25, i100, HMP7, Zymo and CAMI experiments. Strain-level accuracy is measured at the level of individual database genomes. Bracken LAST+MEGAN-LR were not designed to achieve strain-level resolution and Kraken and Kraken 2 were not designed for compositional estimation; the corresponding cells are therefore grayed out. Metric L1 quantifies the difference between the true and inferred composition vectors using the L1 norm; metric r^2 quantifies the similarity between the true and inferred composition vectors using Pearson's r^2; both metrics are limited to columns that are non-0 in either the true or the inferred composition vector. "precisionBinary_avg" and "recallBinary_avg" specify recall and precision on the presence and absence of taxonomic entities at the compositional level, independent of abundance ("binary classification metrics").

Supplementary Data 4. **Identity and genome coverage plots for HMP7.** The file shows summary statistics (read length, estimated alignment identities, and genome coverage for each genome with an estimated frequency >0.1%; alternating colors in the coverage plots indicate chromosome boundaries) for the HMP7 analysis. Note how the *Actinomyces* genome differs from the other genomes in terms of alignment identities and spatial genome coverage. MetaMaps comes with a lightweight R script for the generation of equivalent plots for user datasets.

Supplementary Data 5. Accuracy for limited-memory analysis and with a different length threshold. The table shows read assignment and compositional estimation accuracy metrics on experiments i100 and HMP7, comparing the standard mode of MetaMaps (minimum read length = 1000, no memory limit) with two different parameter settings (increasing the minimum read length to 2000 bases and setting a memory limit of 20 GB). See Supplementary Data 1 and 3 for definitions of the reported metrics and Supplementary Table 1 for an evaluation of the effect of the modified settings on runtime and memory usage.

Supplementary Data 6. Summary of the Zymo data. The Zymo data were generated by sequencing the Zymo Community Standards 2 (Even) mock community on an Oxford Nanopore GridION device, and by randomly sampling 5Gb of the generated reads. To generate a truth set, all reads were mapped using

bwa against Zymo-provided reference genomes. “taxonID” and “Name” specify the NCBI taxonomy ID and name of the organism; “Bases” and “nReads” specify, for each organism, the sum of read lengths and the absolute read count assigned to each organism in the truth set. “Minimum mash distance” specifies the minimum mash distance between the Zymo-provided reference genome and the closest in-database genome.

Supplementary Data 7. Summary of the i100 simulated data. i100 represents a medium-complexity metagenome of 96 species. “taxonID” and “Name” specify the NCBI taxonomy ID and the name of the organism, “NCs” the contig IDs that were used for read simulation with pbsim. “Bases”, “nReads” and “Genomes” refer to the number of simulated bases, reads and genome equivalents per organism.

Supplementary Data 8. Summary of the p25 simulated data. p25 comprises 15 potentially pathogenic and 10 common bacterial species. “taxonID” and “Name” specify the NCBI taxonomy ID and the name of the organism, “NCs” the contig IDs that were used for read simulation with pbsim. “Bases”, “nReads” and “Genomes” refer to the number of simulated bases, reads and genome equivalents per organism.

Supplementary Data 9. Summary of the HMP7 data. The HMP7 data were generated by sequencing a mock community sample generated by the Human Microbiome Project with the PacBio technology. To generate a truth set, all reads were mapped using bwa against the reference genomes specified in the sample product information sheet (column “GIs used for truth-set mapping”). “taxonID” and “Name” specify the NCBI taxonomy ID and name of the organism; “Bases” and “nReads” specify, for each organism, the sum of read lengths and the absolute read count assigned to each organism in the truth set; “Genomes” is the base count divided by approximate genome length.
