# Supplementary material for: Strain-level metagenomic assignment and compositional estimation for long reads with MetaMaps
Source: Nat Commun. 2019 Jul 11;10:3066. doi: 10.1038/s41467-019-10934-2 (PMC6624308; doi:10.1038/s41467-019-10934-2)

MetaMaps mapping summary for Helicobacter pylori 26695−1 (e−proteobacteria) (taxon ID x902) − 31700 mapped reads assigned

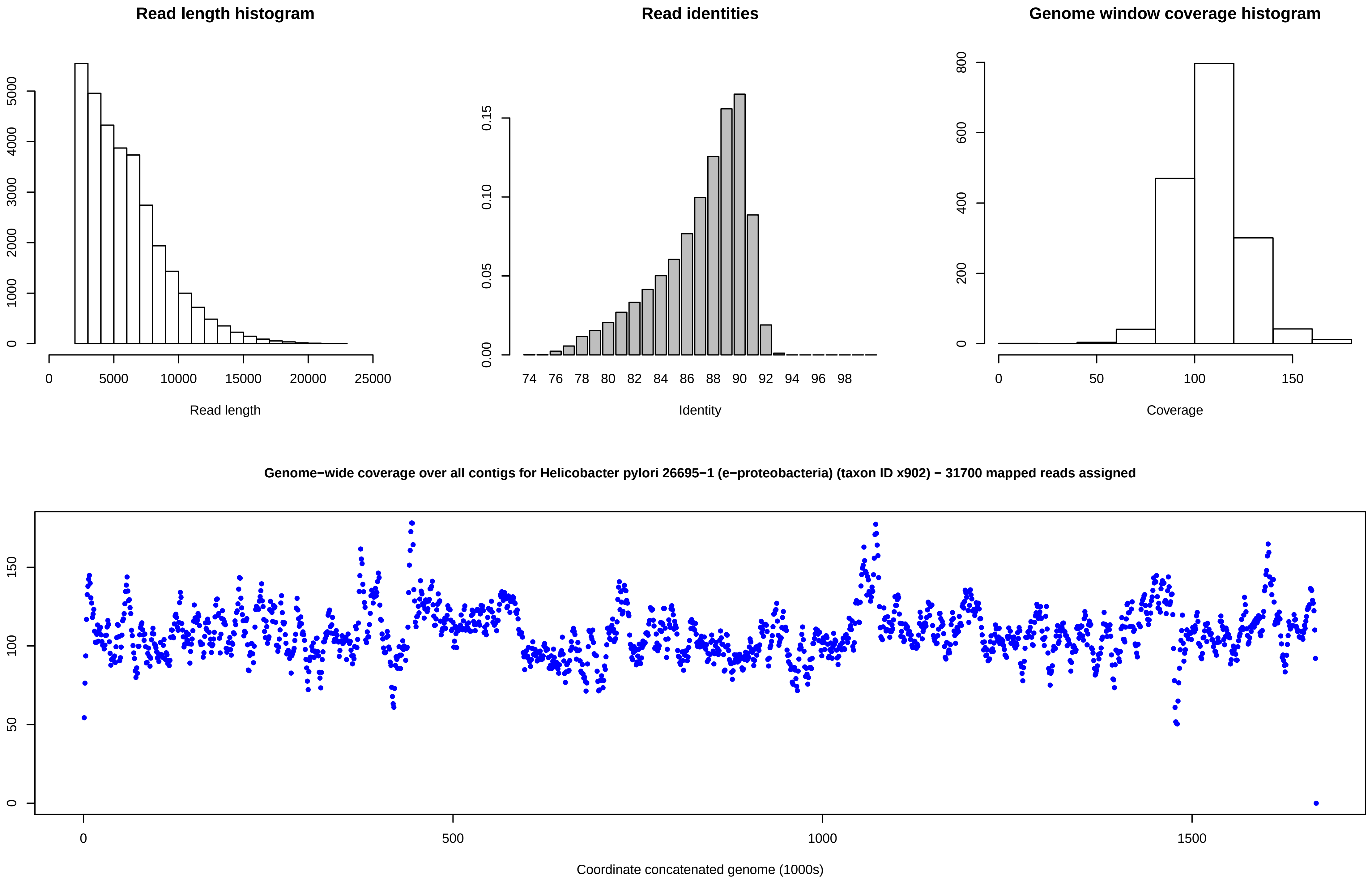

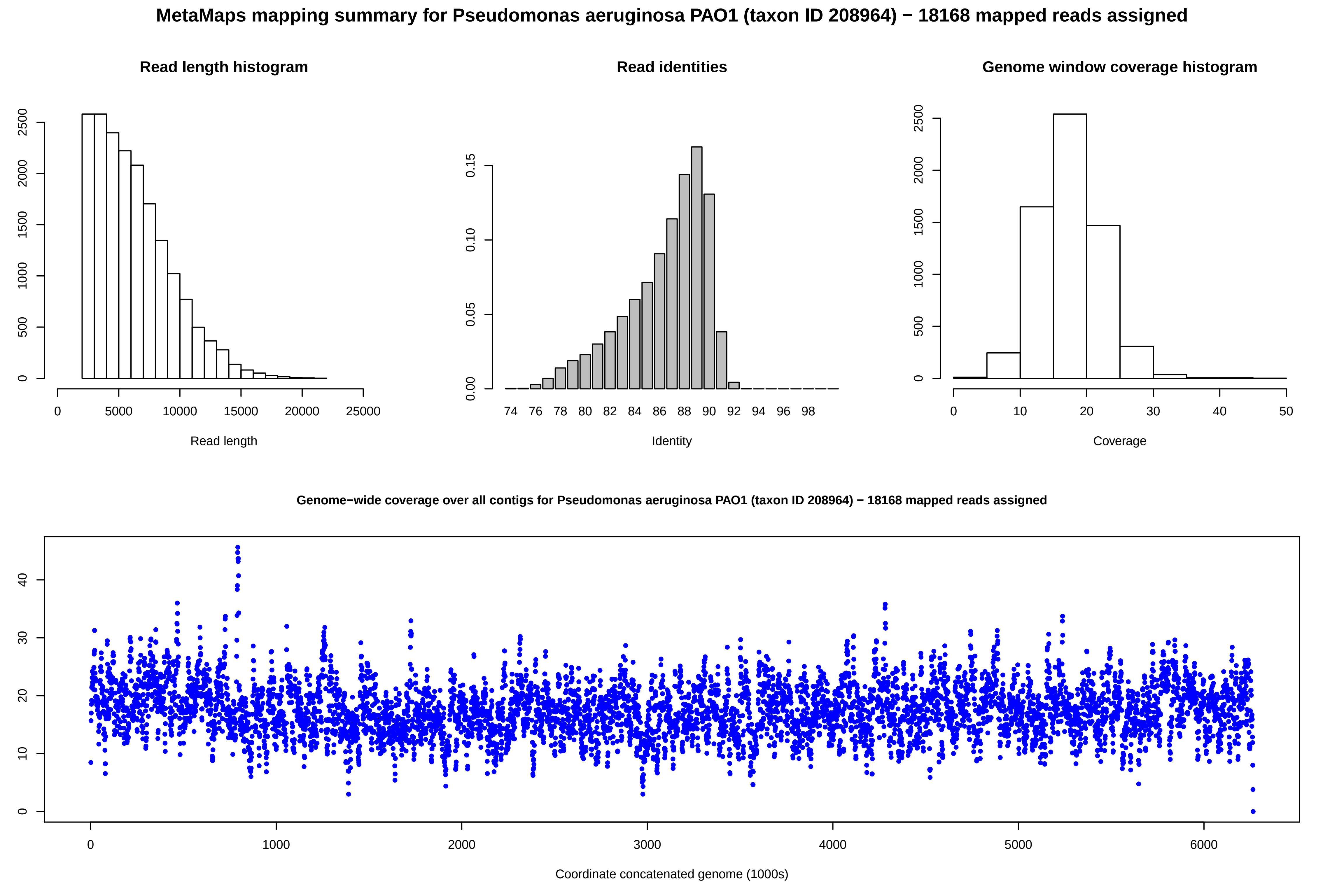
MetaMaps mapping summary for Pseudomonas aeruginosa PAO1 (taxon ID 208964) − 18168 mapped reads assigned
Read length histogram
0
500
1000
1500
2000
2500
0
5000
10000
15000
20000
25000
Read length
Read identities
0.00
0.05
0.10
0.15
74
76
78
80
82
84
86
88
90
92
94
96
98
Identity
Genome window coverage histogram
0
500
1000
1500
2000
2500
0
10
20
30
40
50
Coverage
Genome−wide coverage over all contigs for Pseudomonas aeruginosa PAO1 (taxon ID 208964) − 18168 mapped reads assigned
0
10
20
30
40
0
1000
2000
3000
4000
5000
6000
Coordinate concatenated genome (1000s)

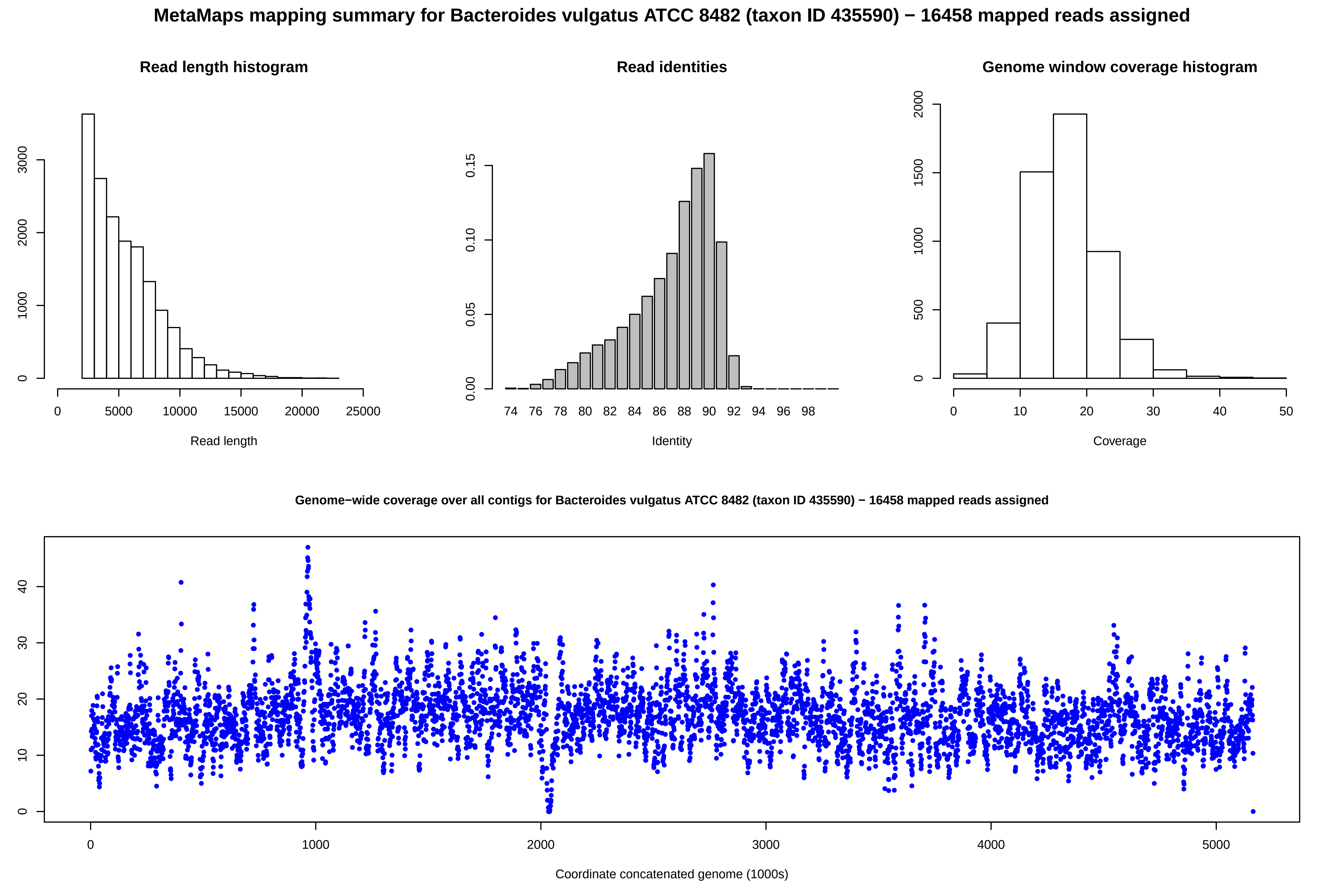
MetaMaps mapping summary for Bacteroides vulgatus ATCC 8482 (taxon ID 435590) − 16458 mapped reads assigned
Read length histogram
Read length
Read identities
Identity
Genome window coverage histogram
Coverage
Genome−wide coverage over all contigs for Bacteroides vulgatus ATCC 8482 (taxon ID 435590) − 16458 mapped reads assigned
Coordinate concatenated genome (1000s)

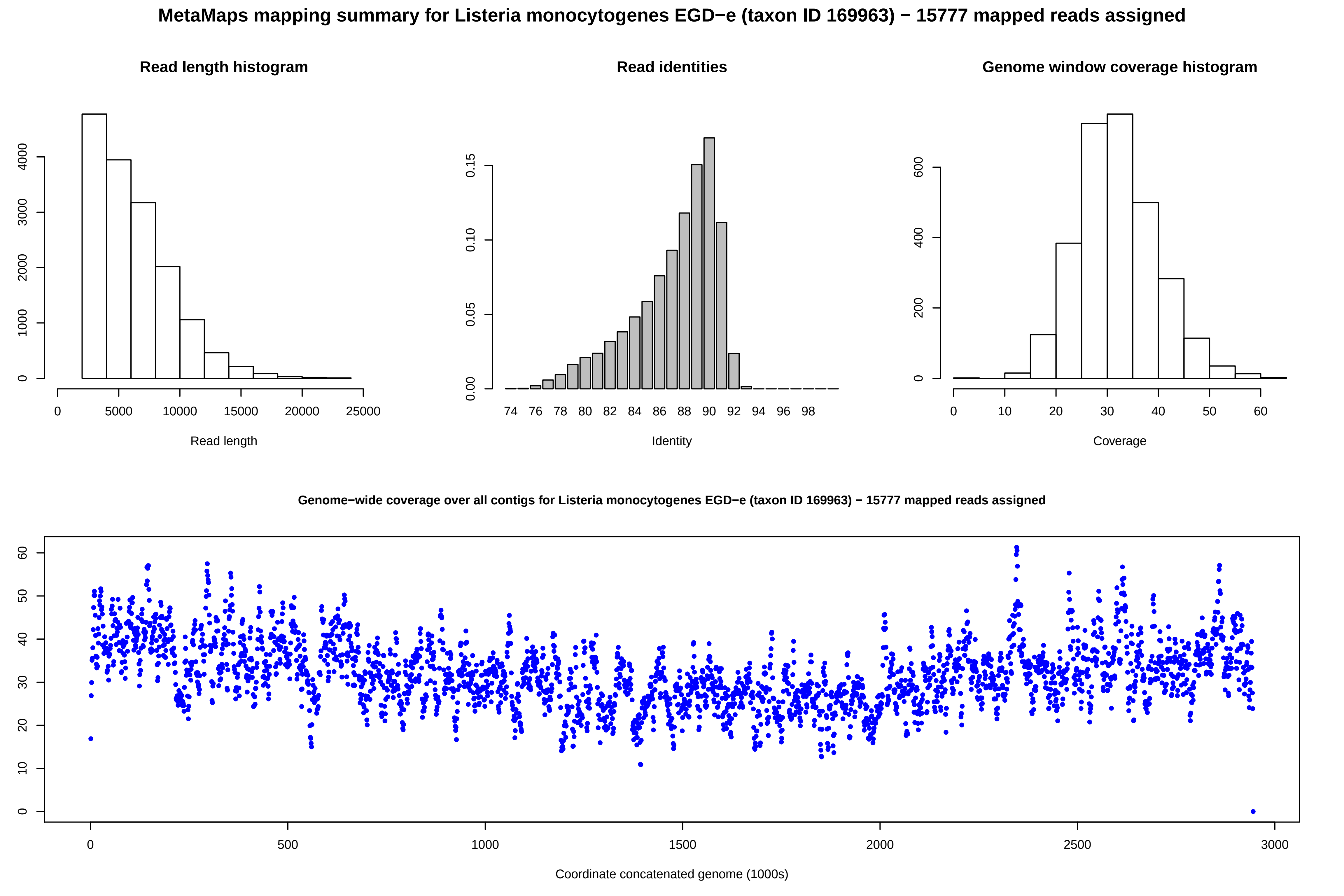

MetaMaps mapping summary for Listeria monocytogenes EGD−e (taxon ID 169963) − 15777 mapped reads assigned
Read length histogram
Read length
Read identities
Identity
Genome window coverage histogram
Coverage
Genome−wide coverage over all contigs for Listeria monocytogenes EGD−e (taxon ID 169963) − 15777 mapped reads assigned
Coordinate concatenated genome (1000s)

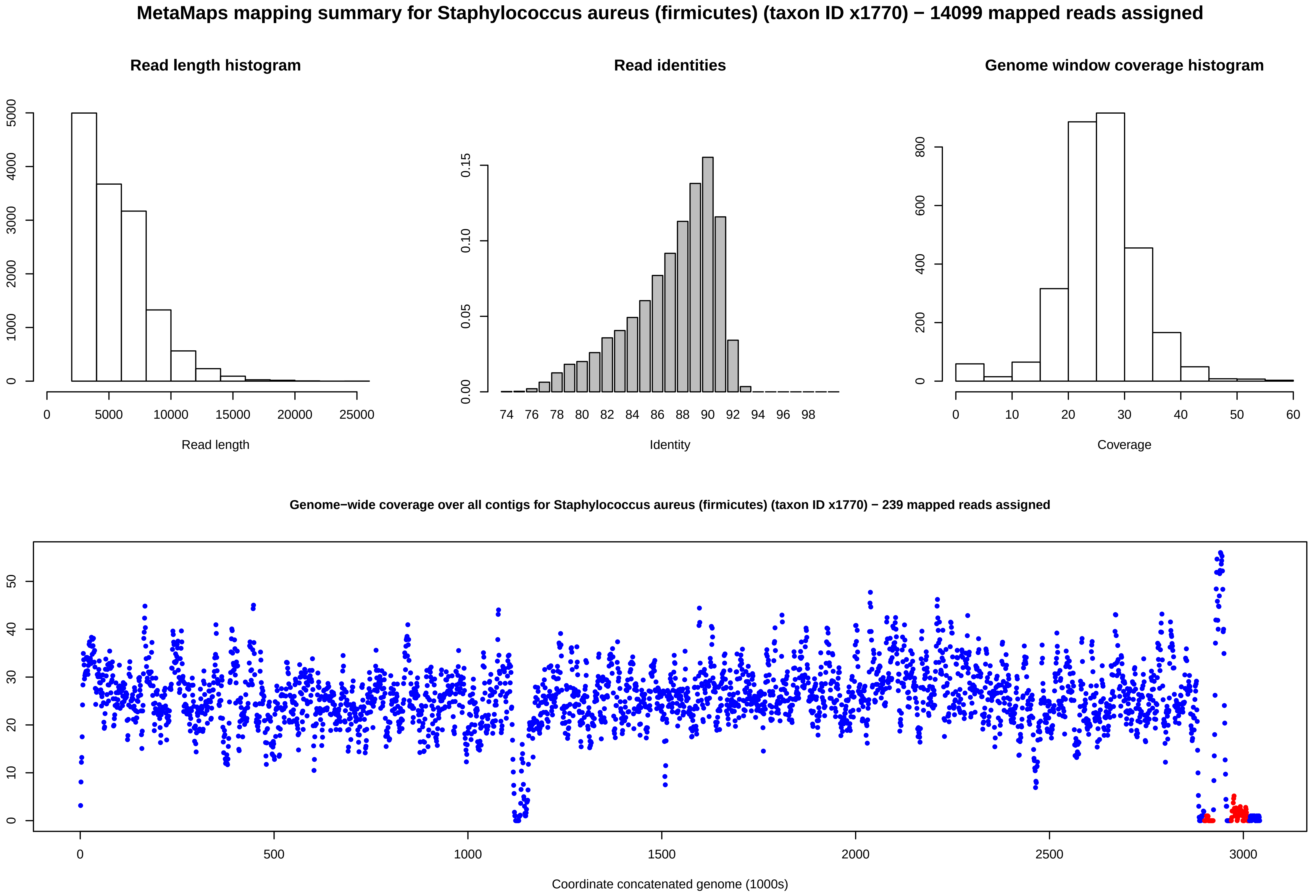
MetaMaps mapping summary for Staphylococcus aureus (firmicutes) (taxon ID x1770) − 14099 mapped reads assigned
Read length histogram
0 1000 2000 3000 4000 5000
0 5000 10000 15000 20000 25000
Read length
Read identities
0.00 0.05 0.10 0.15
74 76 78 80 82 84 86 88 90 92 94 96 98
Identity
Genome window coverage histogram
0 200 400 600 800
0 10 20 30 40 50 60
Coverage
Genome−wide coverage over all contigs for Staphylococcus aureus (firmicutes) (taxon ID x1770) − 239 mapped reads assigned
0 10 20 30 40 50
0 500 1000 1500 2000 2500 3000
Coordinate concatenated genome (1000s)

MetaMaps mapping summary for Clostridium beijerinckii NCIMB 8052 (taxon ID 290402) − 12996 mapped reads assigned

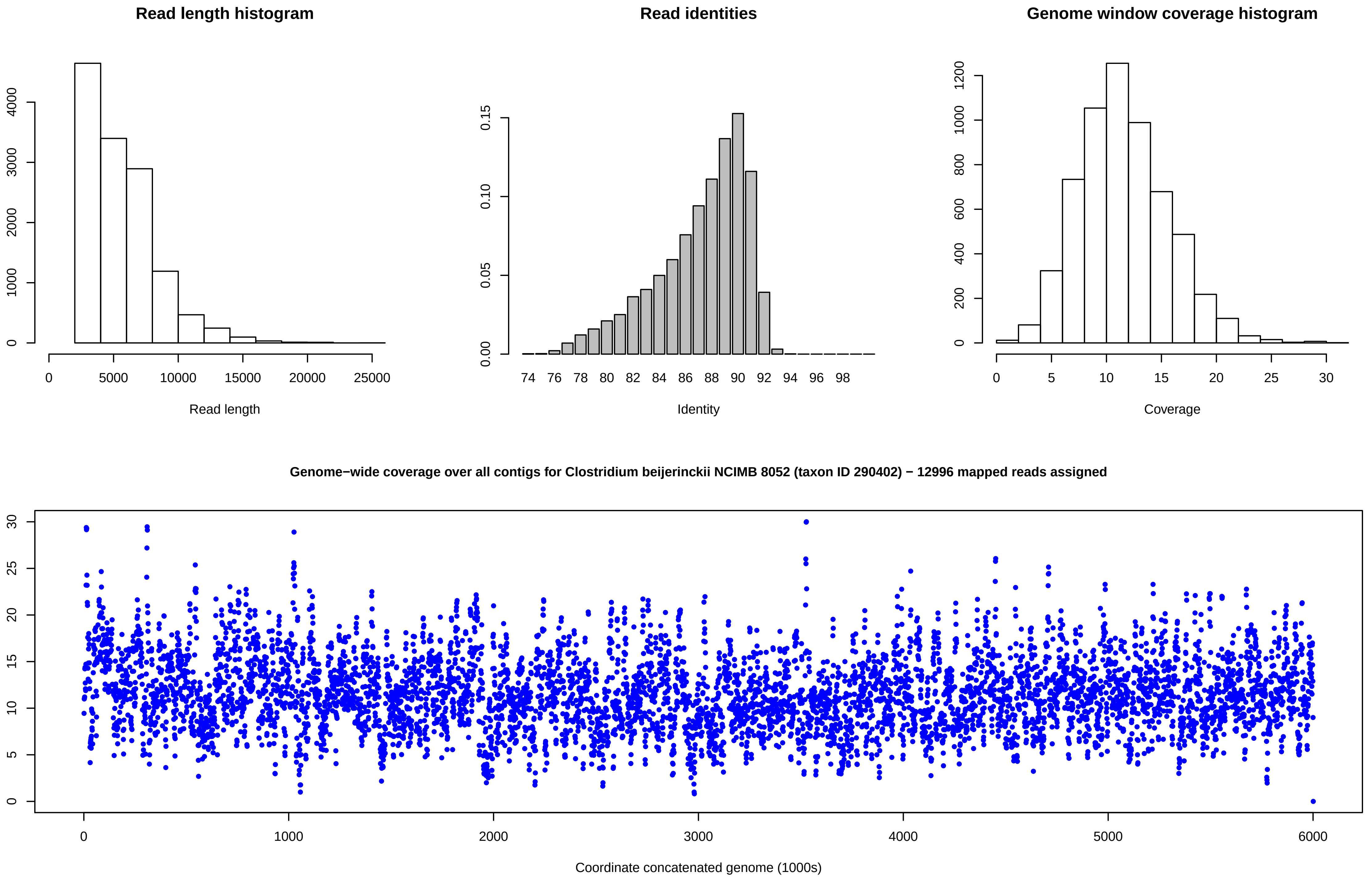

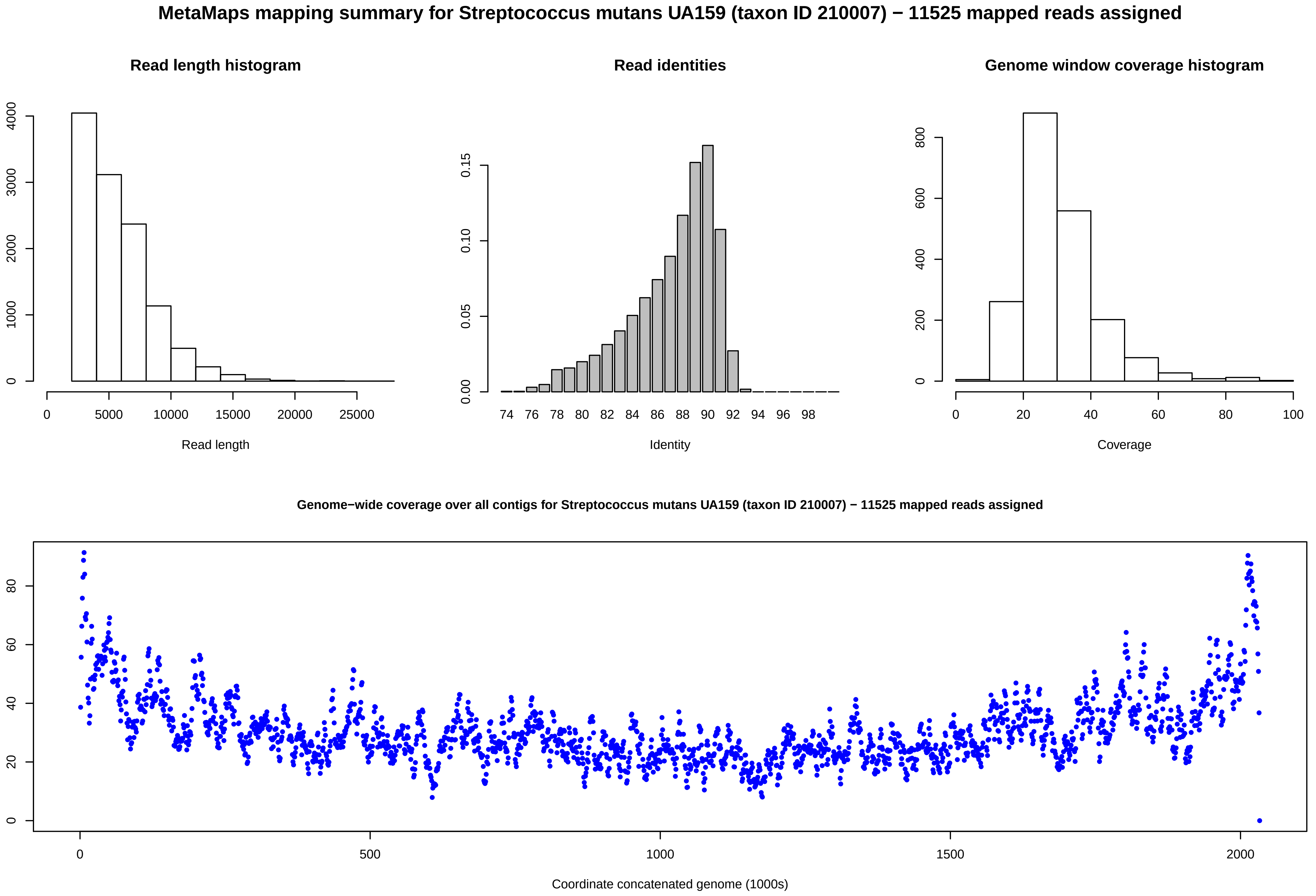

MetaMaps mapping summary for Streptococcus mutans UA159 (taxon ID 210007) − 11525 mapped reads assigned
Read length histogram
Read length
0
5000
10000
15000
20000
25000
0
1000
2000
3000
4000
Read identities
Identity
74
76
78
80
82
84
86
88
90
92
94
96
98
0.00
0.05
0.10
0.15
Genome window coverage histogram
Coverage
0
20
40
60
80
100
0
200
400
600
800
Genome−wide coverage over all contigs for Streptococcus mutans UA159 (taxon ID 210007) − 11525 mapped reads assigned
Coordinate concatenated genome (1000s)
0
500
1000
1500
2000
0
20
40
60
80

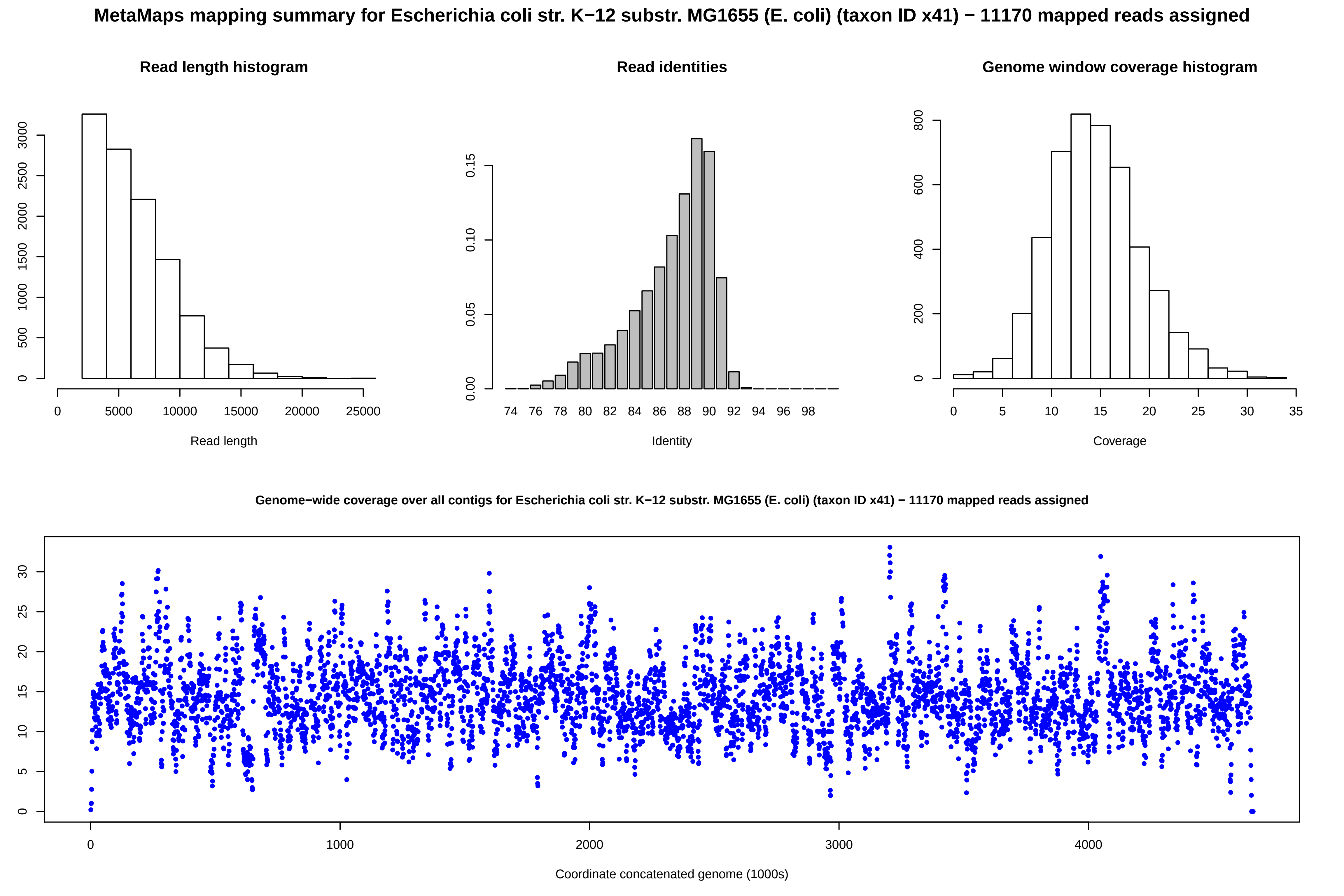
MetaMaps mapping summary for Escherichia coli str. K−12 substr. MG1655 (E. coli) (taxon ID x41) − 11170 mapped reads assigned
Read length histogram
Read length
0
5000
10000
15000
20000
25000
500
1000
1500
2000
2500
3000
Read identities
Identity
74
76
78
80
82
84
86
88
90
92
94
96
98
0.00
0.05
0.10
0.15
Genome window coverage histogram
Coverage
0
5
10
15
20
25
30
35
200
400
600
800
Genome−wide coverage over all contigs for Escherichia coli str. K−12 substr. MG1655 (E. coli) (taxon ID x41) − 11170 mapped reads assigned
Coordinate concatenated genome (1000s)
1000
2000
3000
4000

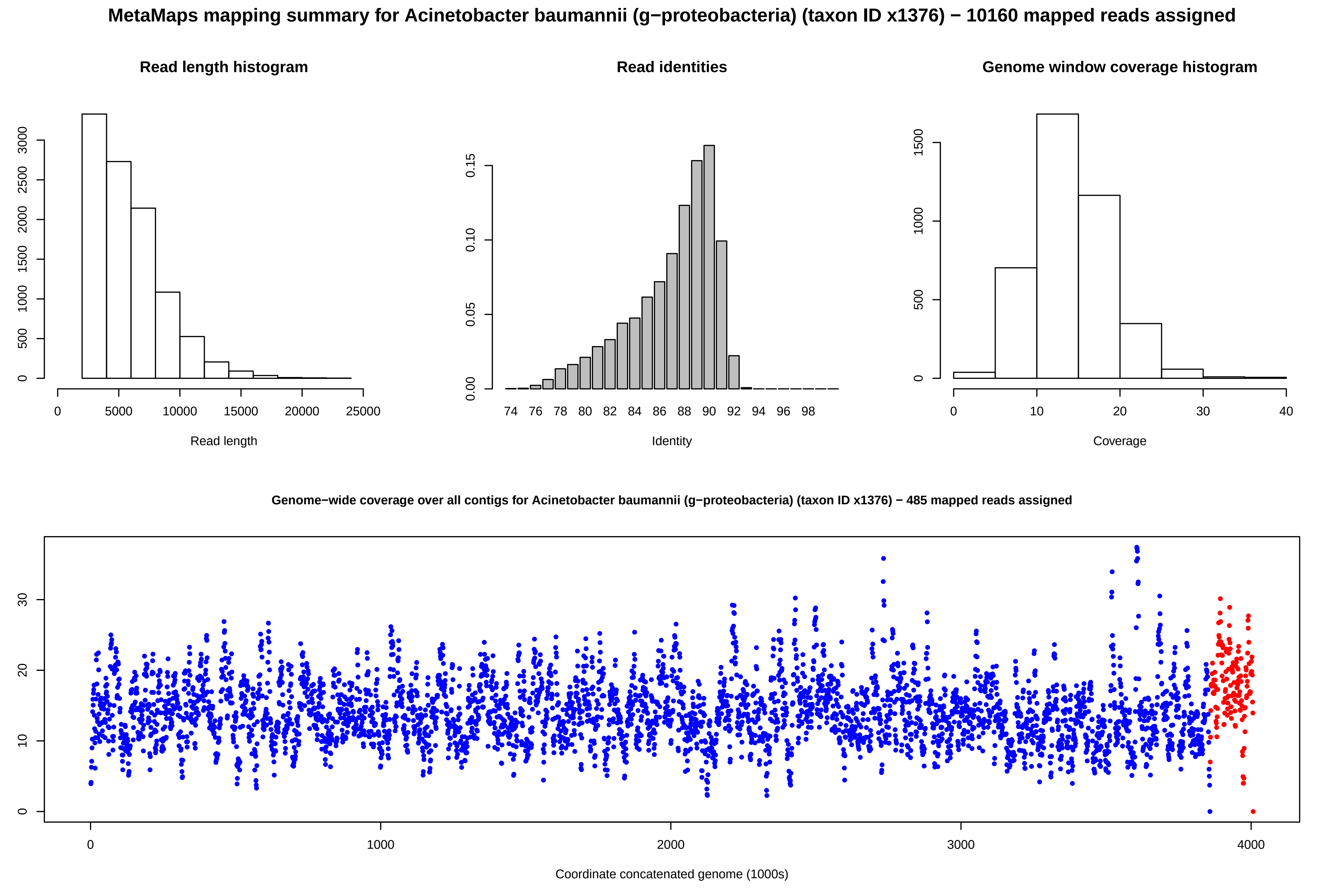
MetaMaps mapping summary for Acinetobacter baumannii (g−proteobacteria) (taxon ID x1376) − 10160 mapped reads assigned
Read length histogram
Read length
Read identities
Identity
Genome window coverage histogram
Coverage
Genome−wide coverage over all contigs for Acinetobacter baumannii (g−proteobacteria) (taxon ID x1376) − 485 mapped reads assigned
Coordinate concatenated genome (1000s)

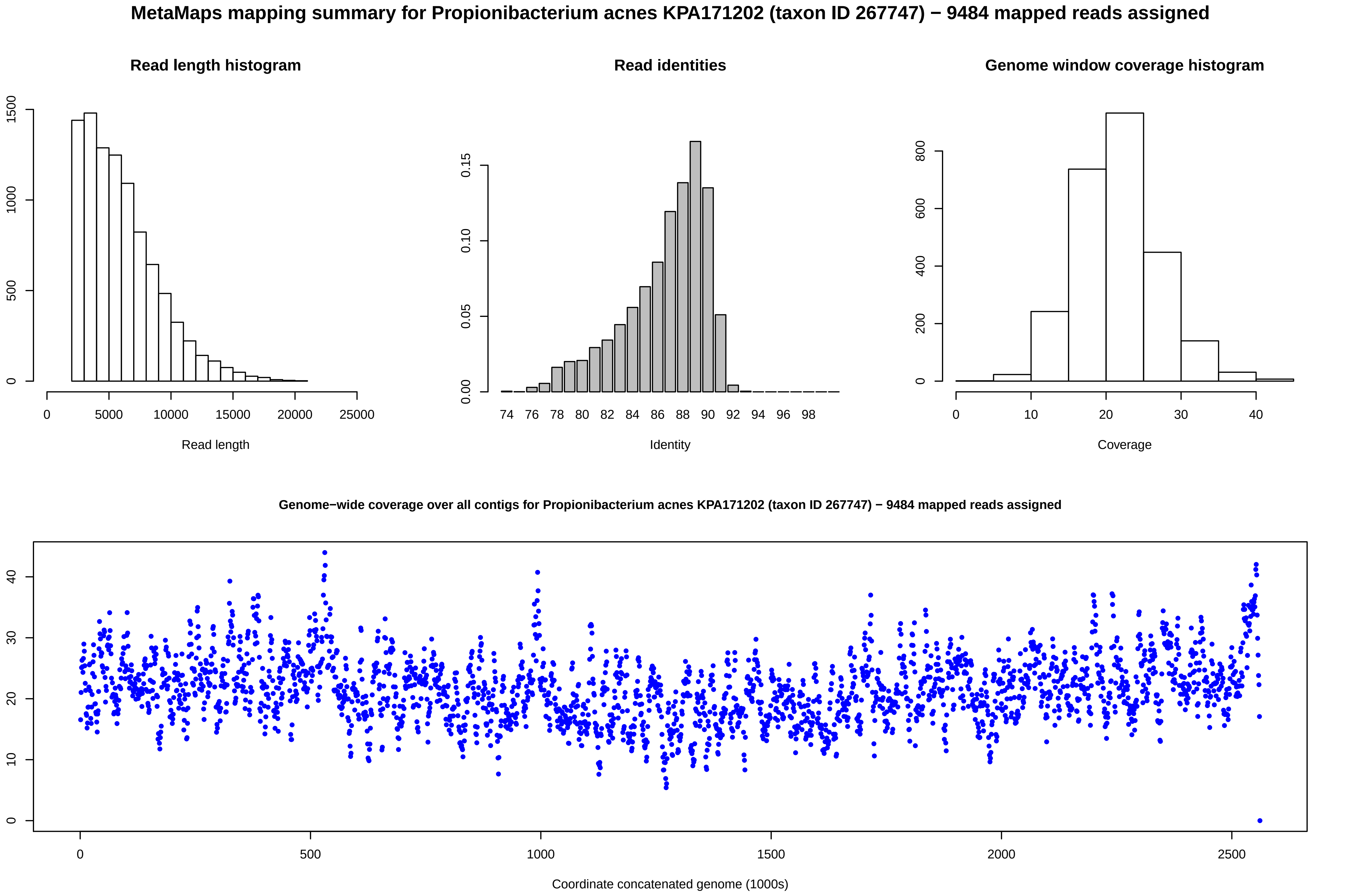
MetaMaps mapping summary for Propionibacterium acnes KPA171202 (taxon ID 267747) − 9484 mapped reads assigned
Read length histogram
0
500
1000
1500
0
5000
10000
15000
20000
25000
Read length
Read identities
0.00
0.05
0.10
0.15
74
76
78
80
82
84
86
88
90
92
94
96
98
Identity
Genome window coverage histogram
0
200
400
600
800
0
10
20
30
40
Coverage
Genome−wide coverage over all contigs for Propionibacterium acnes KPA171202 (taxon ID 267747) − 9484 mapped reads assigned
0
10
20
30
40
0
500
1000
1500
2000
2500
Coordinate concatenated genome (1000s)

MetaMaps mapping summary for Lactobacillus gasseri ATCC 33323 = JCM 1131 (taxon ID 324831) − 9479 mapped reads assigned

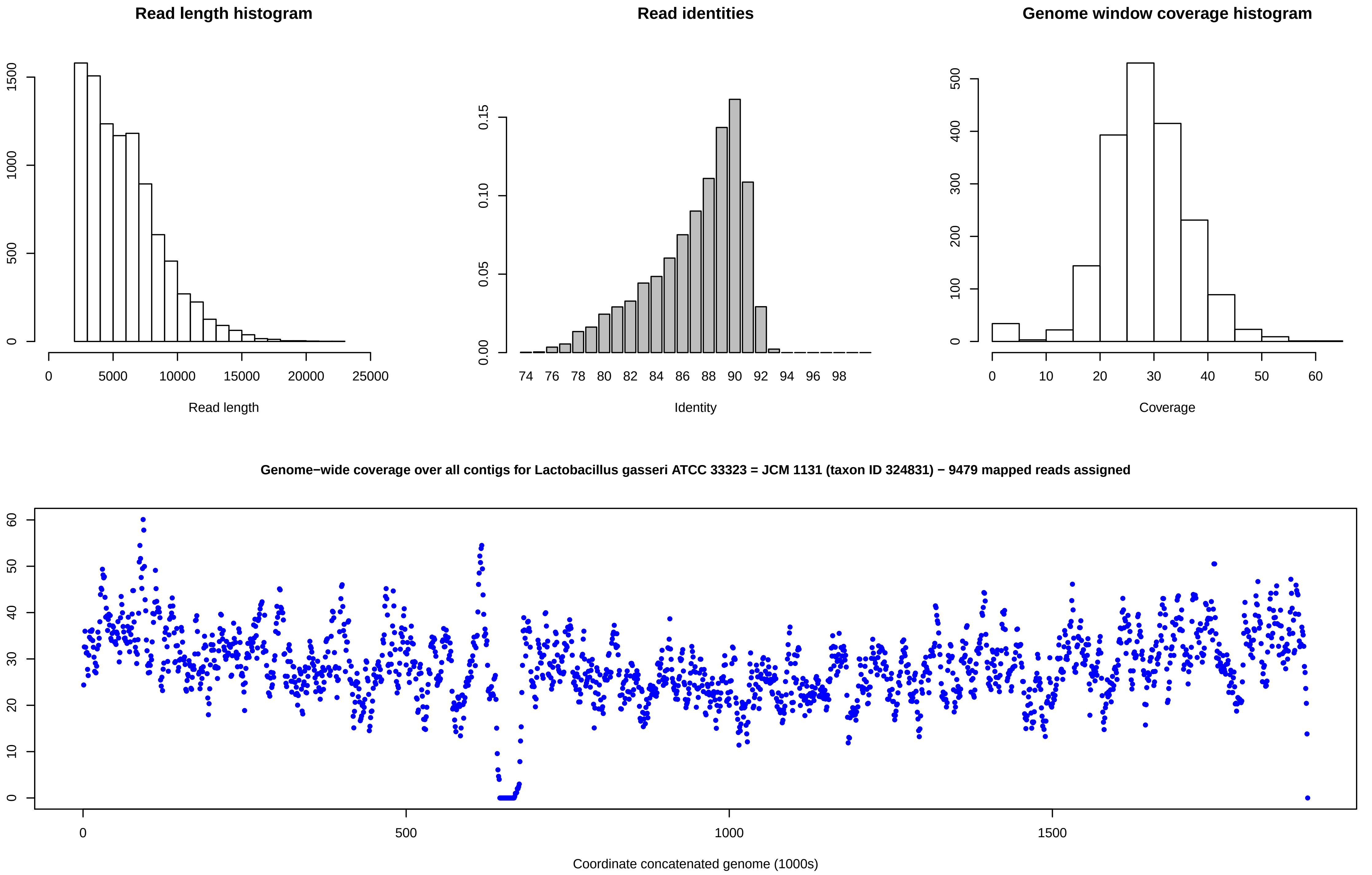

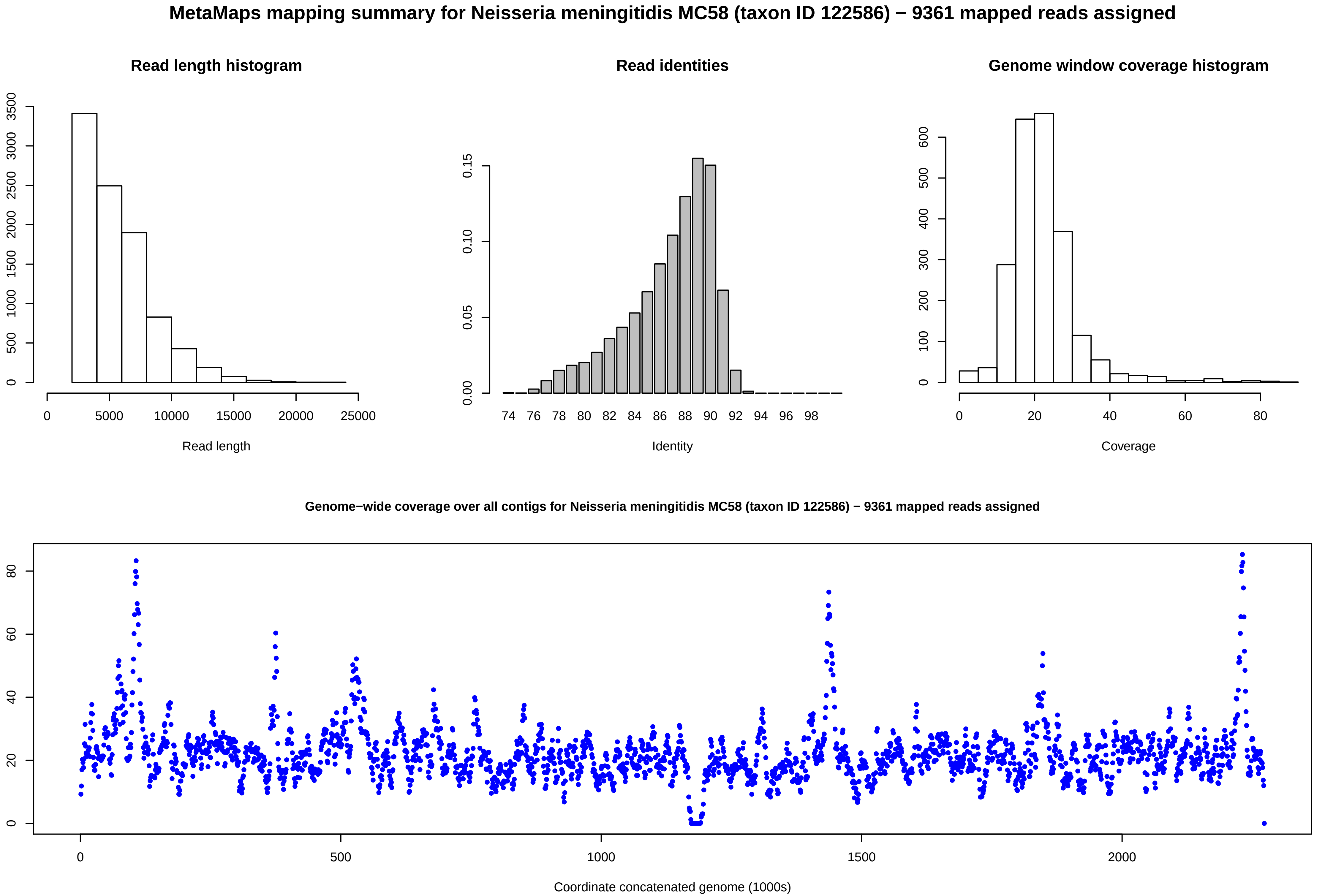
MetaMaps mapping summary for Neisseria meningitidis MC58 (taxon ID 122586) − 9361 mapped reads assigned
Read length histogram
Read length
Read identities
Identity
Genome window coverage histogram
Coverage
Genome−wide coverage over all contigs for Neisseria meningitidis MC58 (taxon ID 122586) − 9361 mapped reads assigned
Coordinate concatenated genome (1000s)

MetaMaps mapping summary for Bacillus cereus ATCC 10987 (taxon ID 222523) − 9769 mapped reads assigned

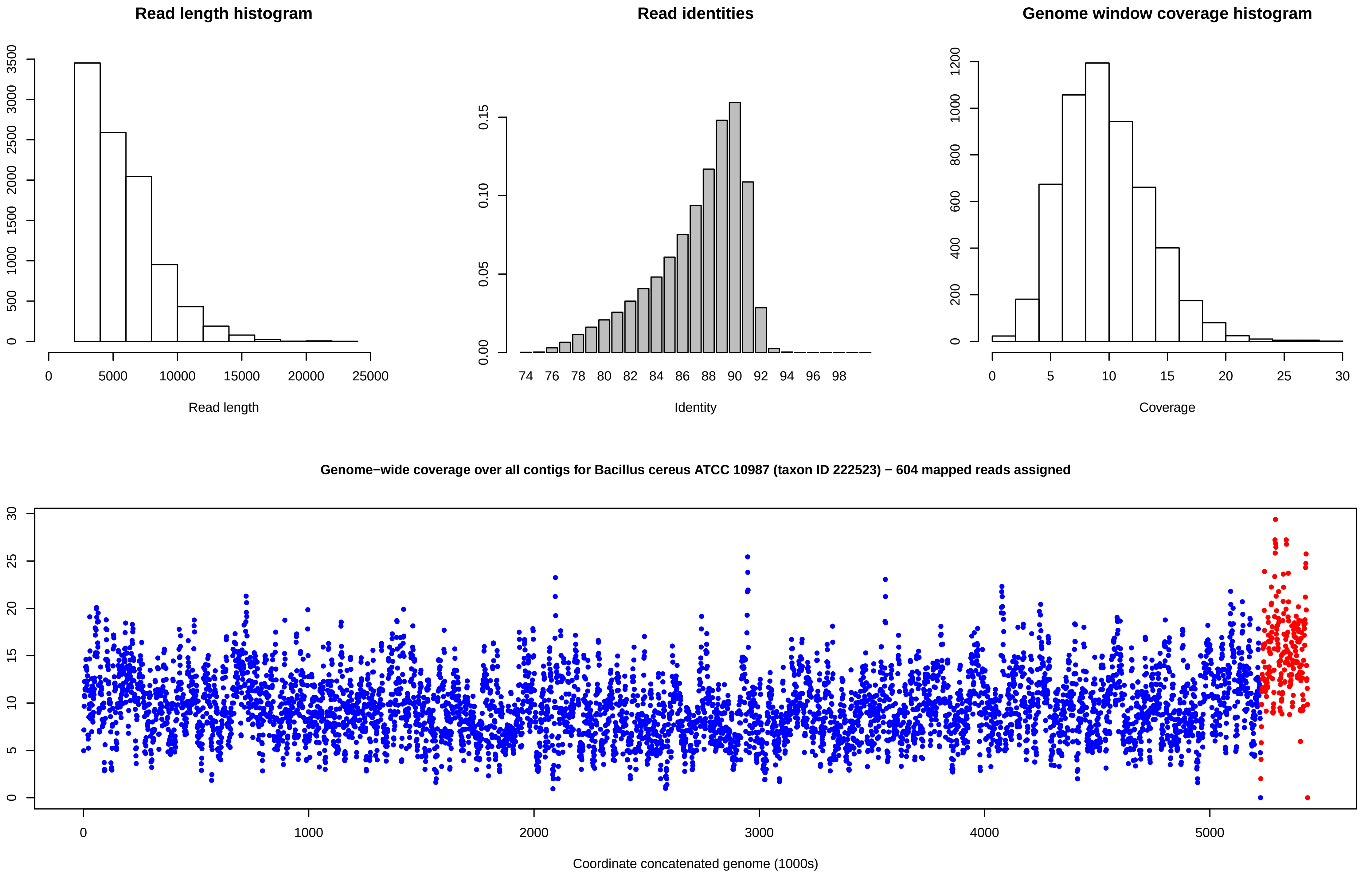

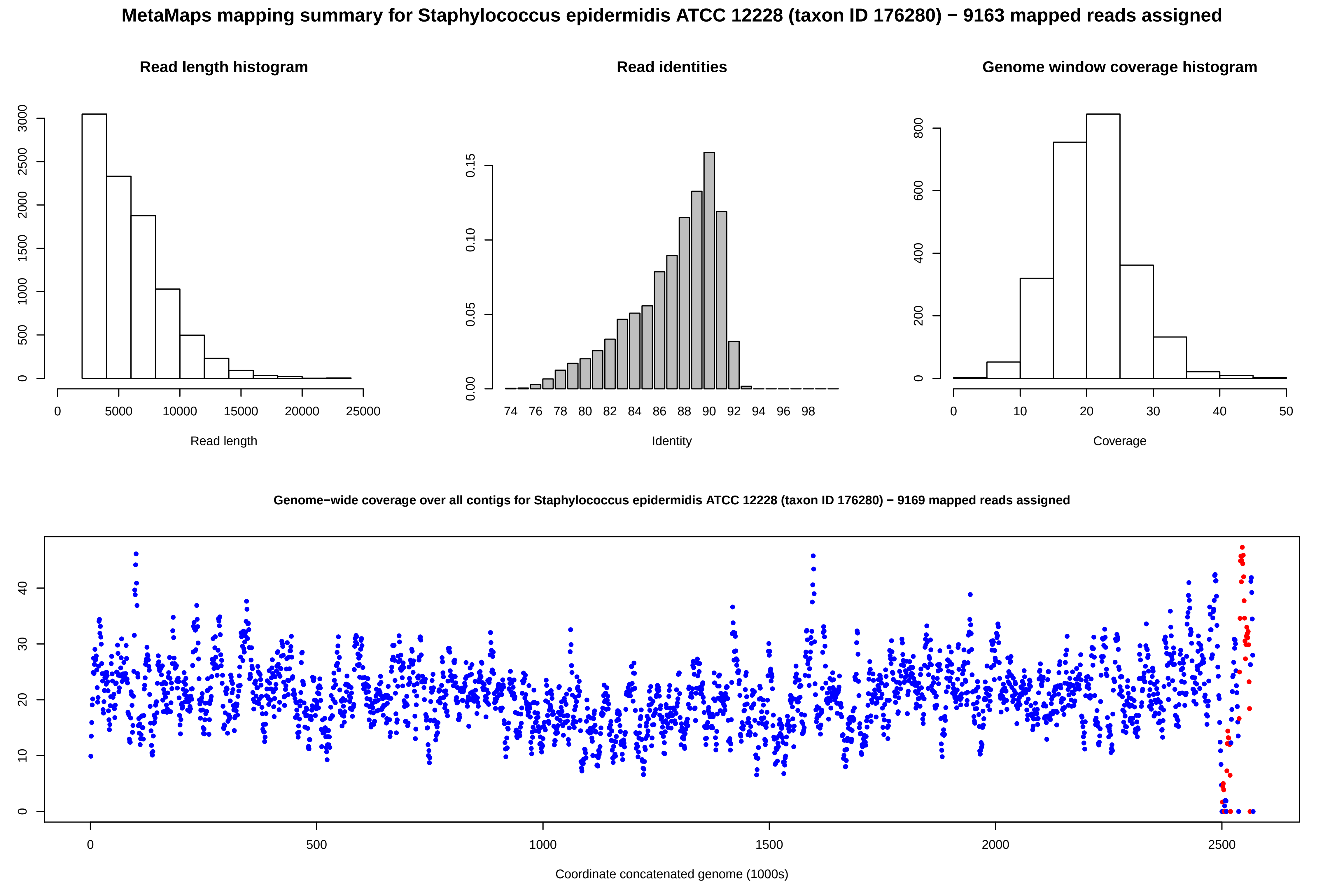
MetaMaps mapping summary for Staphylococcus epidermidis ATCC 12228 (taxon ID 176280) − 9163 mapped reads assigned
Read length histogram
Read length
Read identities
Identity
Genome window coverage histogram
Coverage
Genome−wide coverage over all contigs for Staphylococcus epidermidis ATCC 12228 (taxon ID 176280) − 9169 mapped reads assigned
Coordinate concatenated genome (1000s)

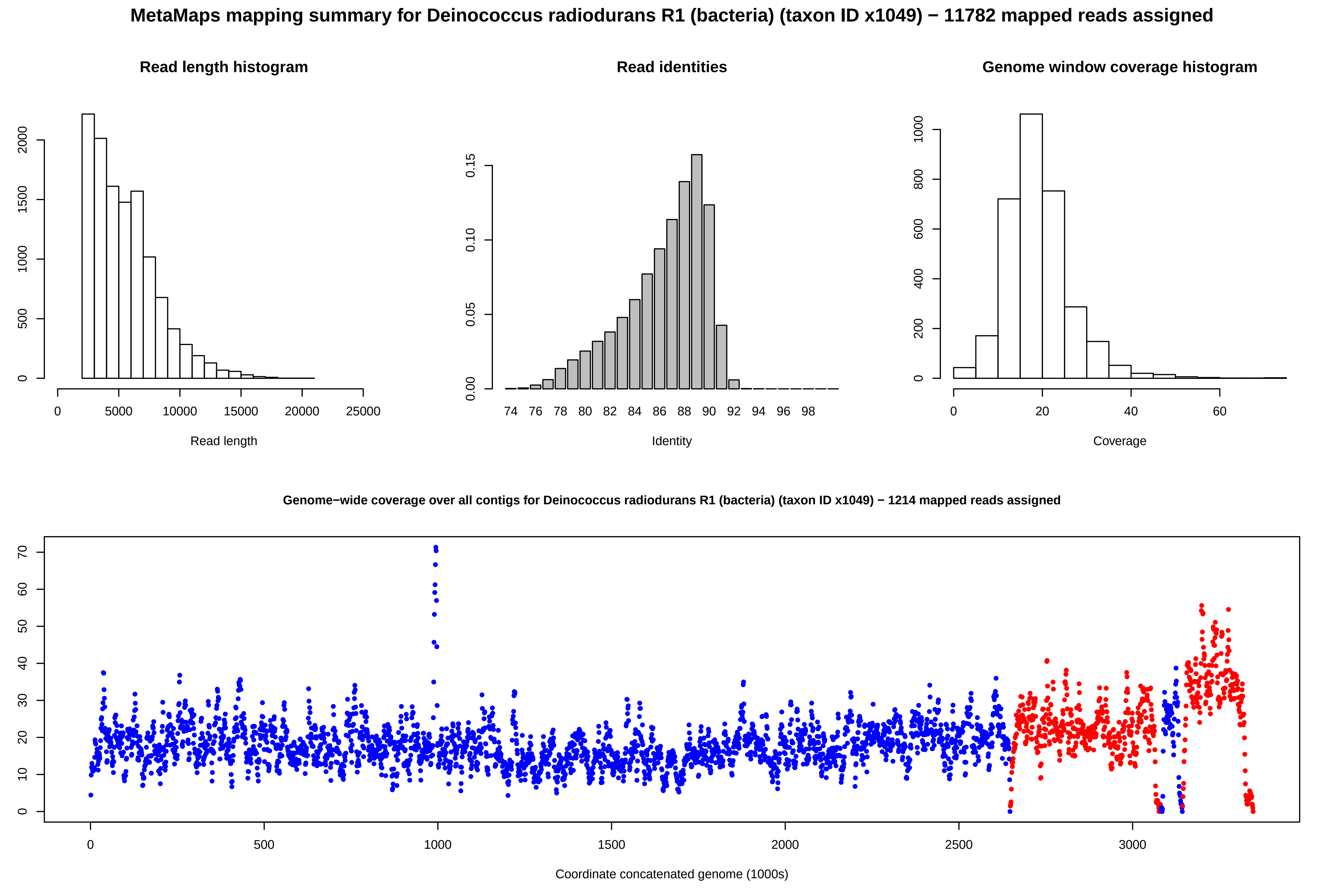

MetaMaps mapping summary for Deinococcus radiodurans R1 (bacteria) (taxon ID x1049) − 11782 mapped reads assigned
Read length histogram
Read length
Read identities
Identity
Genome window coverage histogram
Coverage
Genome−wide coverage over all contigs for Deinococcus radiodurans R1 (bacteria) (taxon ID x1049) − 1214 mapped reads assigned
Coordinate concatenated genome (1000s)

MetaMaps mapping summary for Enterococcus faecalis OG1RF (taxon ID 474186) − 8772 mapped reads assigned

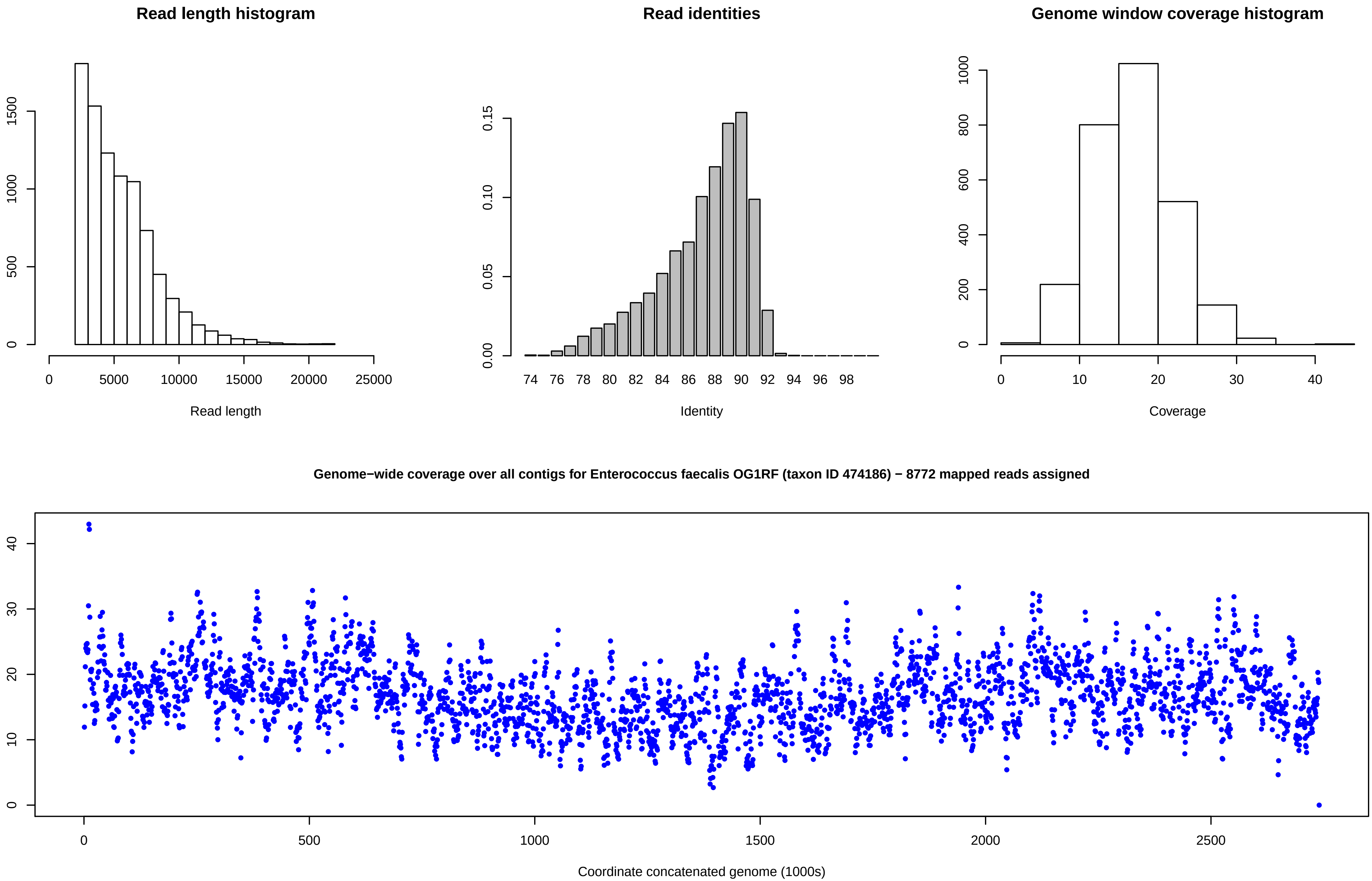

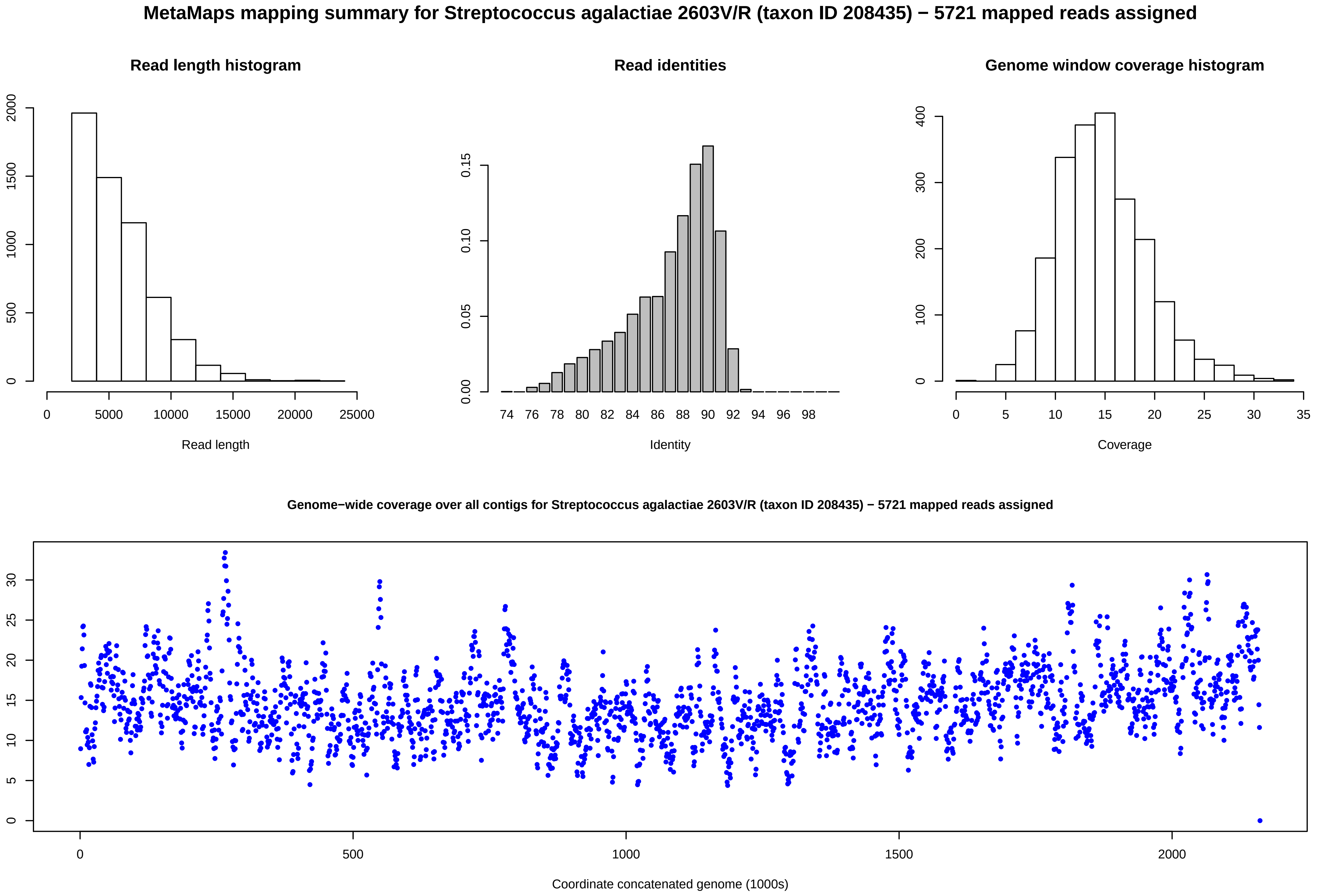
MetaMaps mapping summary for Streptococcus agalactiae 2603V/R (taxon ID 208435) − 5721 mapped reads assigned
Read length histogram
Read length
Read identities
Identity
Genome window coverage histogram
Coverage
Genome−wide coverage over all contigs for Streptococcus agalactiae 2603V/R (taxon ID 208435) − 5721 mapped reads assigned
Coordinate concatenated genome (1000s)

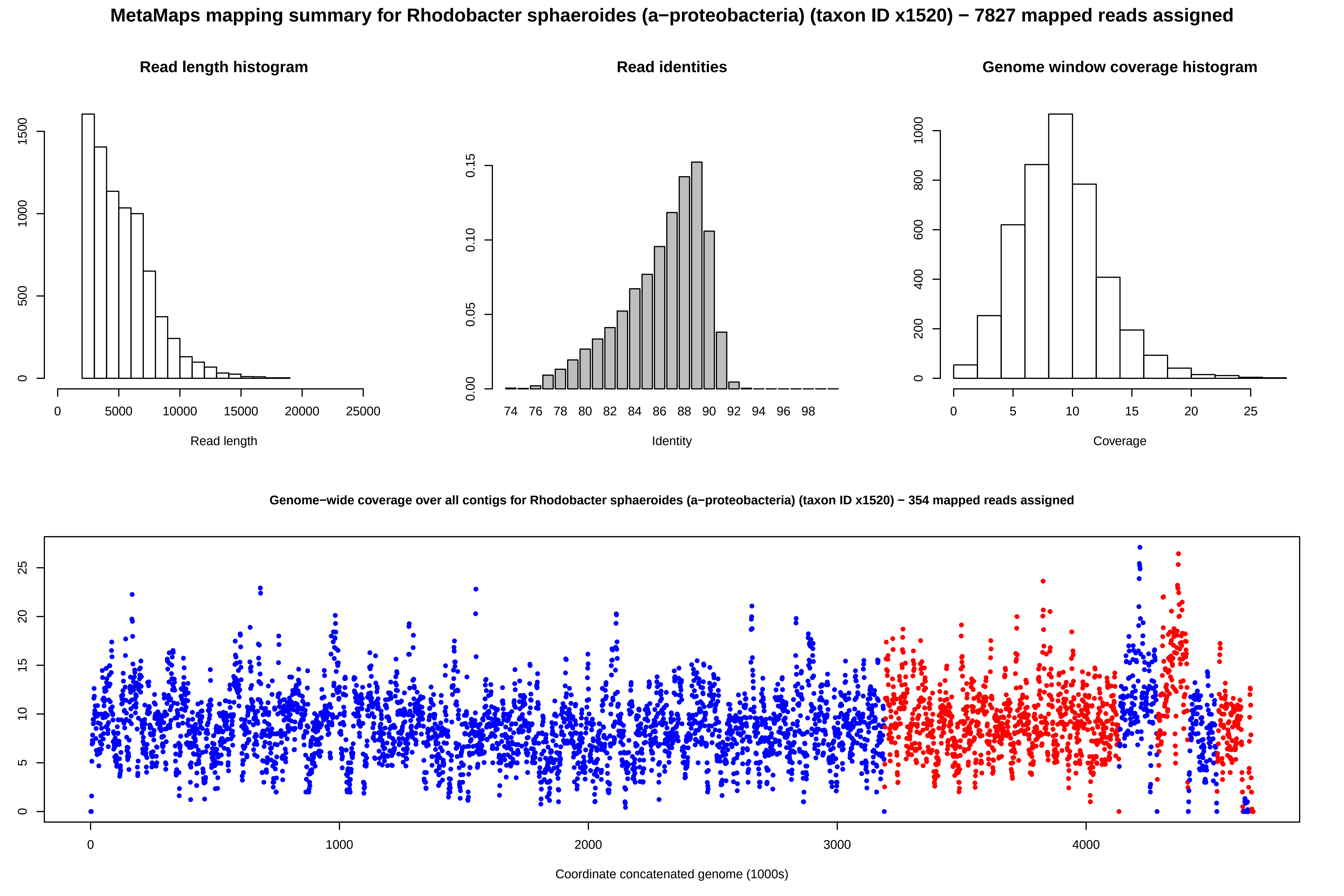
MetaMaps mapping summary for Rhodobacter sphaeroides (a−proteobacteria) (taxon ID x1520) − 7827 mapped reads assigned
Read length histogram
Read length
Read identities
Identity
Genome window coverage histogram
Coverage
Genome−wide coverage over all contigs for Rhodobacter sphaeroides (a−proteobacteria) (taxon ID x1520) − 354 mapped reads assigned
Coordinate concatenated genome (1000s)

MetaMaps mapping summary for Streptococcus pneumoniae TIGR4 (taxon ID 170187) − 1326 mapped reads assigned

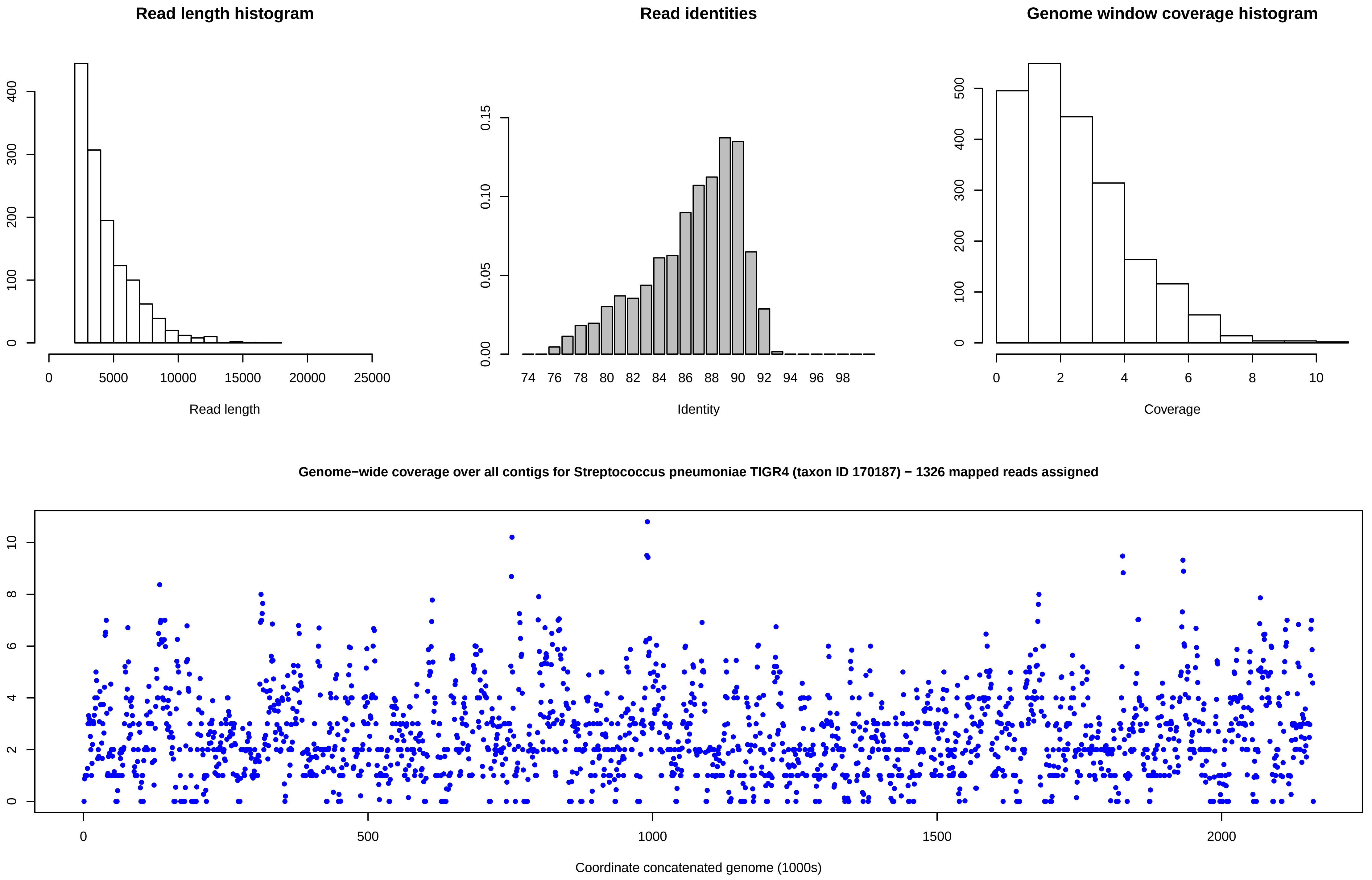

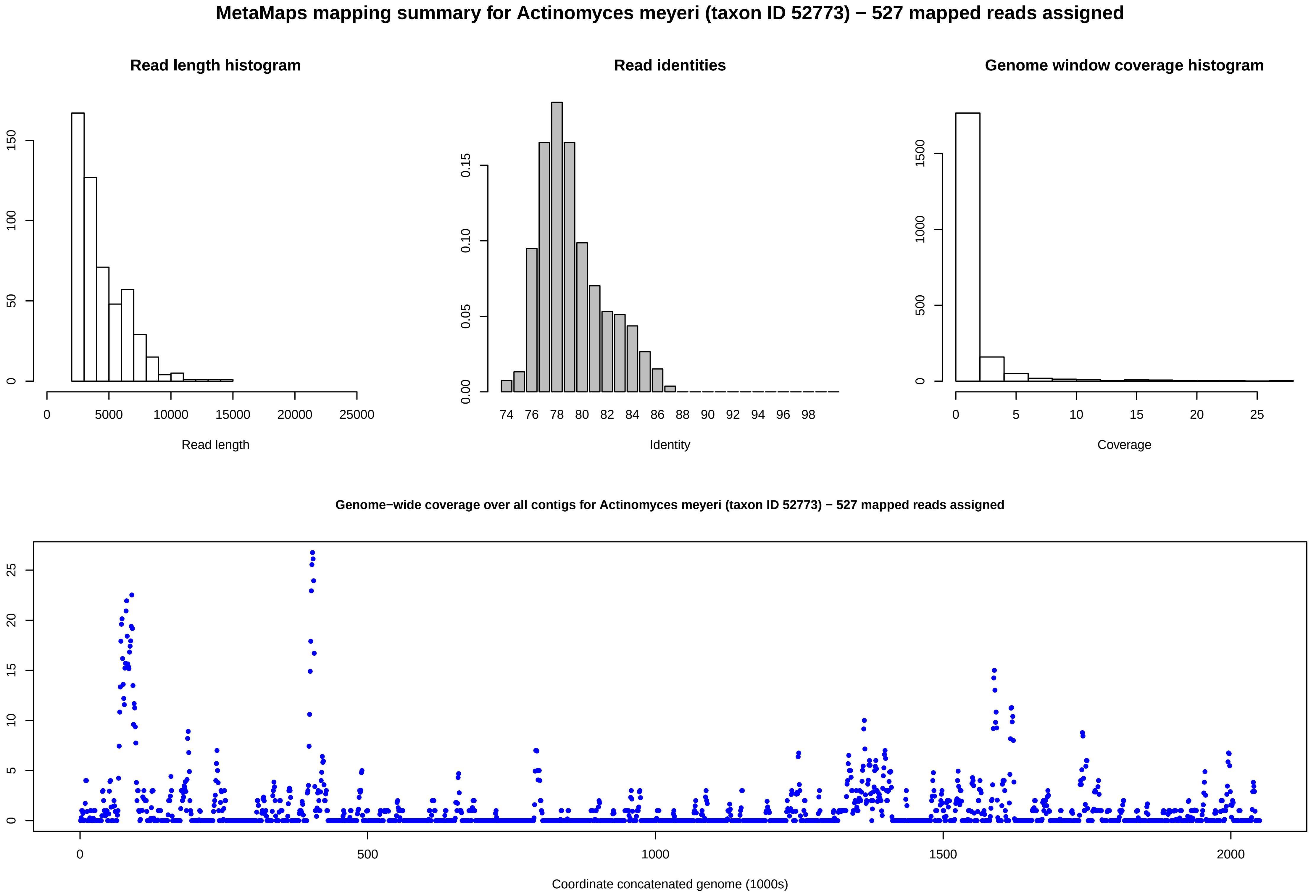
MetaMaps mapping summary for Actinomyces meyeri (taxon ID 52773) − 527 mapped reads assigned
Read length histogram
Read length
Read identities
Identity
Genome window coverage histogram
Coverage
Genome−wide coverage over all contigs for Actinomyces meyeri (taxon ID 52773) − 527 mapped reads assigned
Coordinate concatenated genome (1000s)

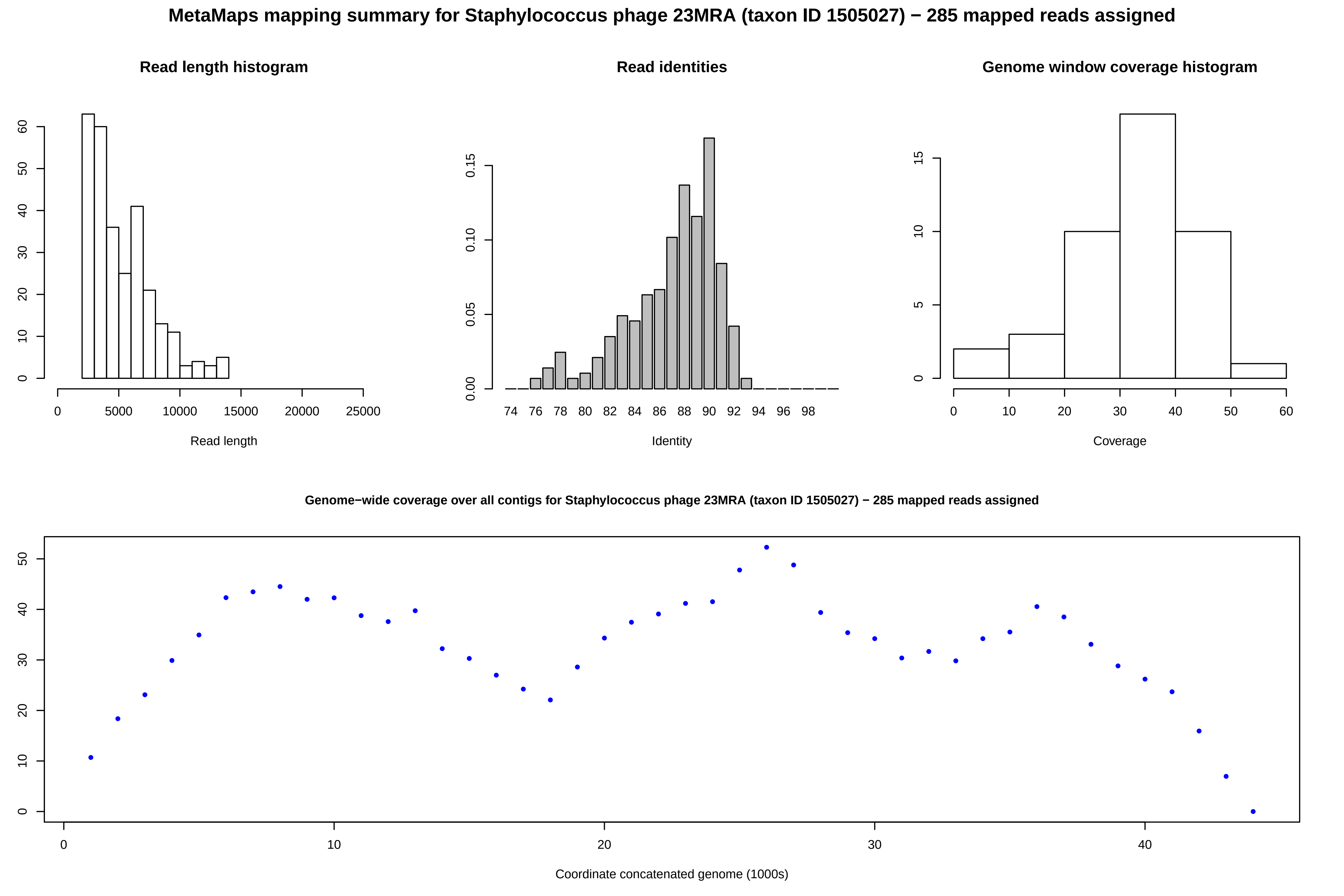
MetaMaps mapping summary for Staphylococcus phage 23MRA (taxon ID 1505027) − 285 mapped reads assigned
Read length histogram
Read length
Read identities
Identity
Genome window coverage histogram
Coverage
Genome−wide coverage over all contigs for Staphylococcus phage 23MRA (taxon ID 1505027) − 285 mapped reads assigned
Coordinate concatenated genome (1000s)

MetaMaps mapping summary for Acinetobacter baumannii AYE (taxon ID 509173) − 267 mapped reads assigned

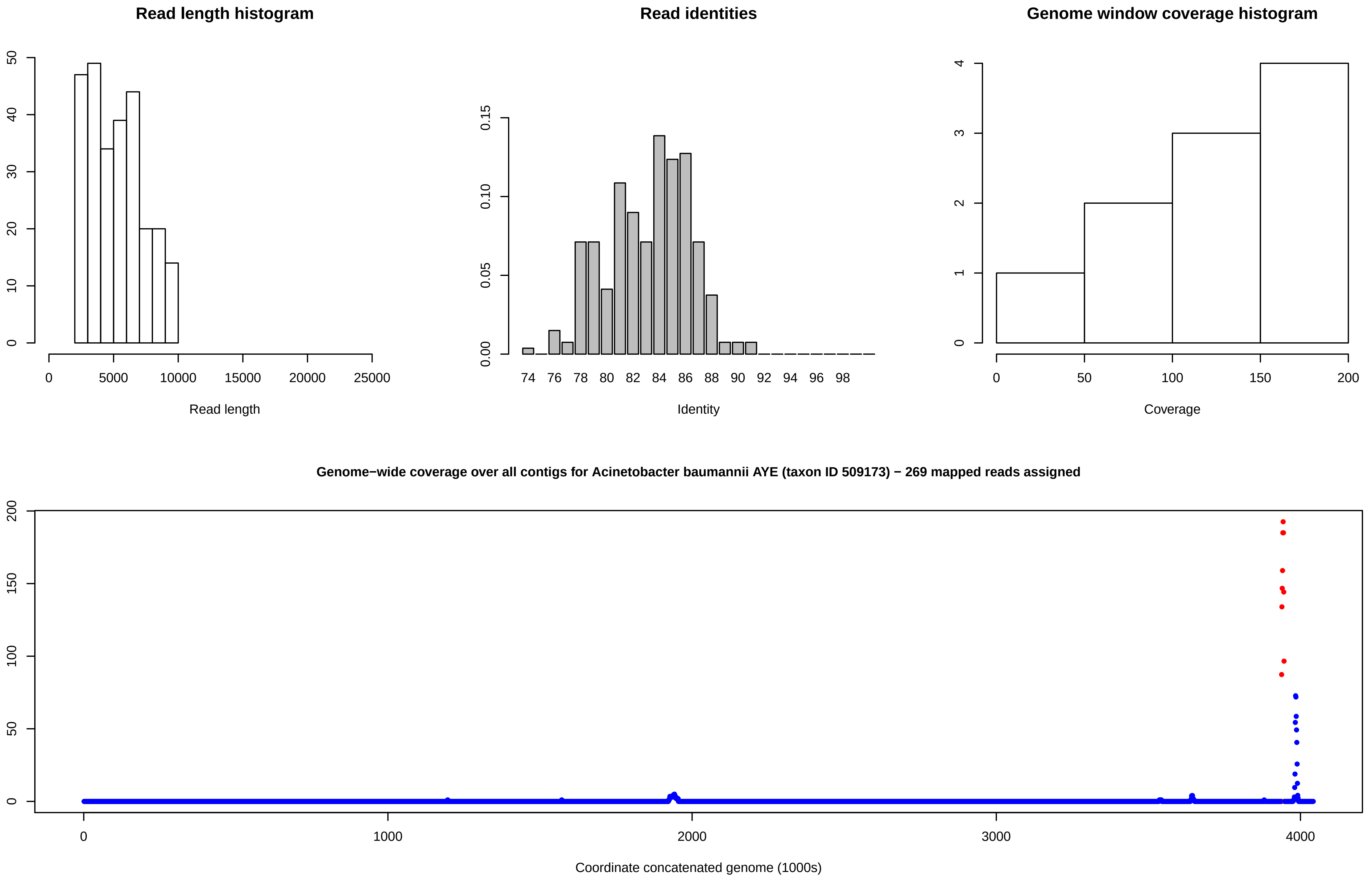

Supplement: Supplementary file 8 — Supplementary Data 4 [file 41467_2019_10934_MOESM8_ESM.pdf]
